# Diagnostic tests for titanium hypersensitivity in implant dentistry: a systematic review of the literature

**DOI:** 10.1186/s40729-022-00428-0

**Published:** 2022-07-11

**Authors:** Lena Katharina Müller-Heupt, Eik Schiegnitz, Sebahat Kaya, Elisabeth Jacobi-Gresser, Peer Wolfgang Kämmerer, Bilal Al-Nawas

**Affiliations:** 1 Department of Oral and Maxillofacial Surgery, Plastic Surgery, University Medical Centre of the Johannes Gutenberg-University Mainz, Augustusplatz 2, 55131 Mainz, Germany; 2 Oral Surgery, Private Practice, Heidesheimer Straße 20, 55124 Mainz, Germany

**Keywords:** Allergy, Dental implant, Genetic predisposition, Genotyping, Hypersensitivity, Implant intolerance, LTT, MELISA, Pro-inflammatory cytokines, Titanium, Titanium dioxide, Tribocorrosion

## Abstract

**Purpose:**

There are rising concerns about titanium hypersensitivity reaction regarding dental endosseous implants. This review aims to summarize and compare the validity and reliability of the available dermatological and laboratory diagnostic tests regarding titanium hypersensitivity. The following PICO design was used: In Patients with titanium dental implants (P) does epicutaneous testing (ECT) (I), compared to lymphocyte transformation test (LTT) or Memory Lymphocyte Immunostimulation Assay (MELISA) (C) detect hypersensitivity reactions (O)? A literature search was performed including all studies dealing with this topic. Studies regarding orthopedic implants were excluded.

**Methods:**

Three databases (MEDLINE PubMed, Cochrane Library, SciELO) were screened for suitable studies and an additional manual search was also performed. Literature regarding hypersensitivity reactions in orthopedic implants, hypersensitivity reactions regarding implants not related to dental or maxillofacial surgery, animal studies and in vitro studies were excluded. A quality assessment of all selected full-text articles was performed. Randomized, controlled trials were evaluated with the Cochrane Risk of Bias Tool I. Cohort studies were assessed according to the New Castle–Ottawa Scale and case series according to Moga et al. (Development of a quality appraisal tool for case series studies using a modified Delphi technique. 2012).

**Results:**

10 studies were included in the quantitative synthesis and available for the endpoint diagnostics of intolerance reactions to titanium dental implants: 2 clinical studies, 7 cohort studies and 1 case series. The potential for bias (internal validity) for these studies was overall rated as high.

**Conclusions:**

The study of the available literature regarding ECT and MELISA or LTT in patients with suspected titanium hypersensitivity showed inconsistent results in terms of reliability and validity and thus, those tests should be regarded cautiously. There is strong evidence that titanium hypersensitivity in dental implants is associated with innate immunity: unspecific pro-inflammatory responses due to particle induced hyperreactivity of macrophages or toxicological responses especially towards nanoparticles rather than activation of the adaptive immune system. Therefore, tests detecting allergies do not seem expedient and inflammatory clinical signs should be regarded as leading parameters.

**Graphical Abstract:**

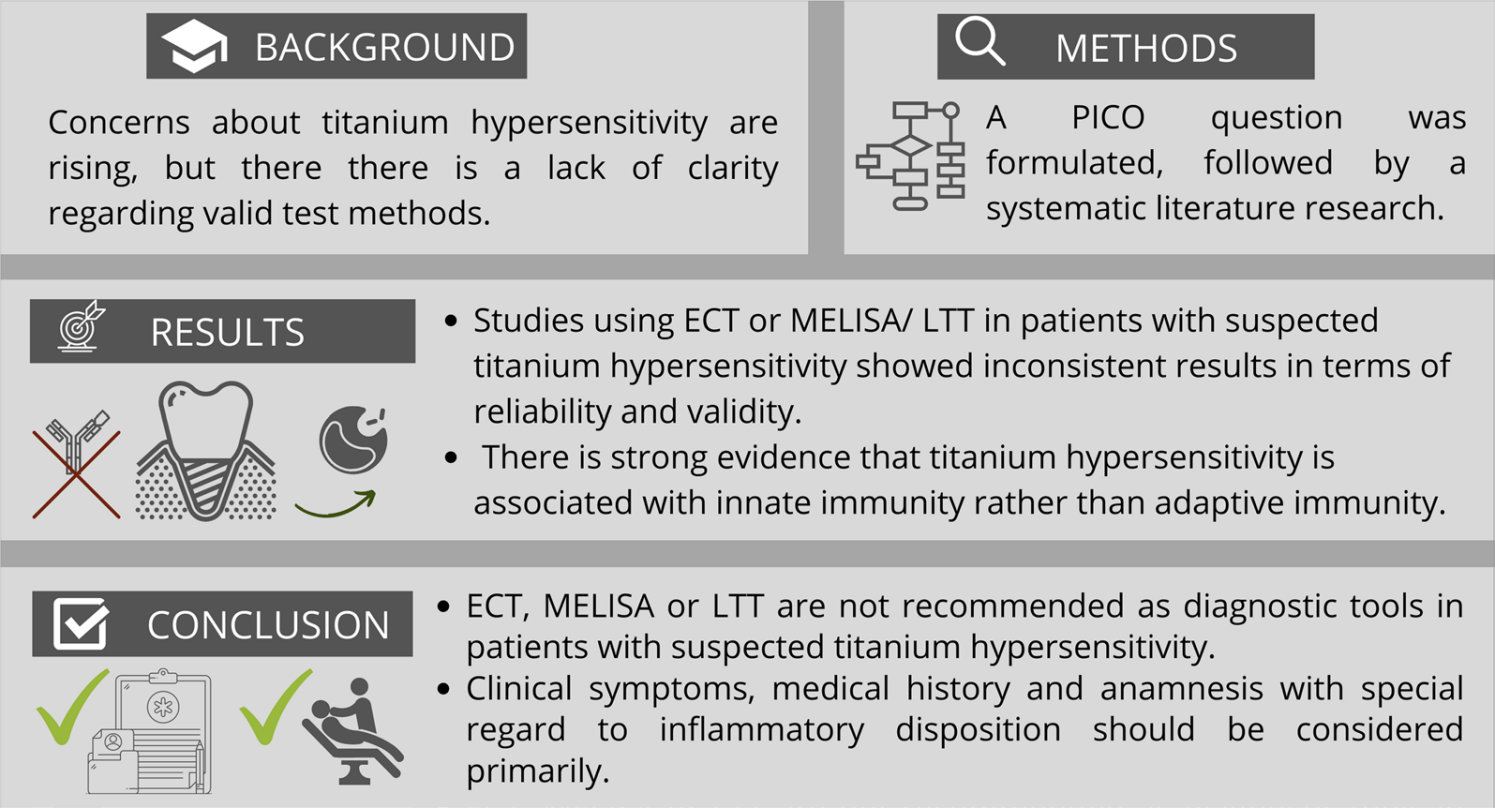

## Introduction

According to the current state of scientific evidence, the risk of damage to health from dental materials is classified as low. Nevertheless, there are concerns regarding intolerance reactions of dental metals causing health impairments. A quarter of randomly chosen people believed that their health was harmed by amalgam fillings containing mercury [1]. Though mercury is still the most infamous dental metal, there is evidence for rising concern in regard to titanium dental implants [2–6].

Intolerance reaction to pure titanium implants (grade 4) is more likely a pro-inflammatory reactivity of tissue macrophages in contact with titanium oxide particles disseminated into the peri-implant tissue by tribocorrosion rather than a systemic allergic reaction. When exposed to oxygen, titanium oxidizes and immediately forms a 1–2 nm thick layer of titanium dioxide (TiO_2_), which protects the titanium surface from further redox reactions [7]. Oxides in general cannot bind to proteins and, therefore, do not function as haptens; they have no allergic potential [8, 9] and additionally a high corrosion resistance [10].

Toxicological studies regarding TiO_2_ revealed adverse effects predominantly mediated by oxidative stress leading to pro-inflammatory responses [11]. The observed adverse effects were dependent on numerous chemical and physical characteristics of the TiO_2_ particles, such as size, crystal structure, specific surface area, particle shape, purity, surface charge and solubility. Intratracheal administered TiO_2_ particles were uptaken by lung-surface macrophages and enhanced recruitment of inflammatory cells leading to tissue damage was observed [12, 13]. A dose dependent mechanism was reported for pulmonary inflammation, fibrosis and epithelial hyperplasia [14]. TiO_2_ particles may contribute significantly to local immune responses [15] and are capable of strongly upregulating pro-inflammatory cytokines such as TNF-α and IL-1β secreted by activated macrophages and monocytes [16]. Those cytokines are primarily associated with innate immunity. Furthermore, toxicity studies of TiO_2_ nanoparticles showed signs of apoptosis and necrosis at certain concentrations [17].

Particles in the peri-implant tissue become phagocytized by macrophages, causing a proinflammatory response by activation of NLRP3 inflammasomes, which is substantially enhanced in the presence of microbial stimuli. Inflammasomes are large intracellular multiprotein complexes that play a central role in innate immunity by activating the release of proinflammatory cytokines [18].

**Fig. 1:**
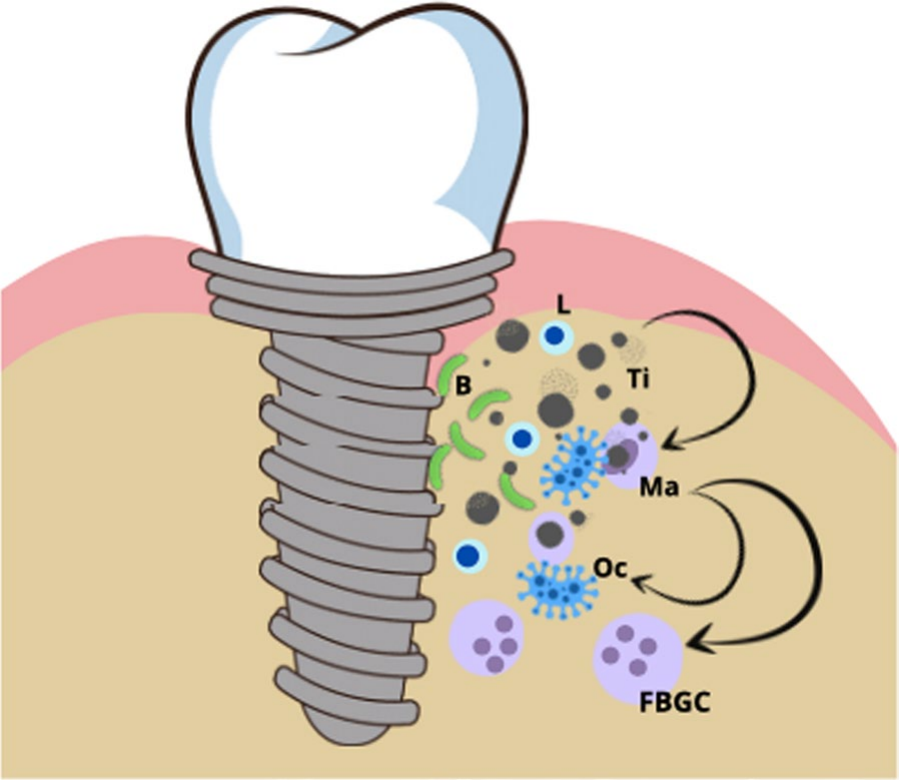
Disruption of the immunological equilibrium and hypothesized immunological cascade of titanium hypersensitivity. Titanium particles trigger a cascade of inflammatory reactions. Macrophages are activated and recruited. Lymphocytes enhance macrophage adhesion and fusion on material surfaces. Macrophages activate osteoclasts and fuse into FBGC leading to bone resorption and secondary bacterial invasion. *L* lymphocyte, *B* bacteria, *Ma* macrophage, *Oc* osteoclasts, *FBGC* Foreign Body Giant Cell

Titanium particles were found in concentrations between 100 and 300 ppm in the peri-implant tissue [19]. Lymphocytes seem to enhance macrophage adhesion and fusion to foreign material surfaces [20]. Bacterial metabolic products create an acidic milieu and increase the release of corrosion wear from the implant surface. It has been convincingly shown that particle load triggers a pro-inflammatory reaction in the peri-implant soft and hard tissues [21–23]. Phagocytizing small titanium dioxide particles (< 10 µm), macrophages are activated and produce pro-inflammatory cytokines [24, 25]. The intensity of a pro-inflammatory reaction most likely depends on individual predispositions. The inflammatory cascade may lead to the breakdown of the foreign body equilibrium (Fig. 1). Activated Macrophages trigger osteoclasts or fuse into foreign body multinucleated giant cells, which dissolve bone mineral fractions [26]. The pathomechanism of inflammasome activation in macrophages by lipoproteins of periodontal pathogens as well as titanium particles is described [58] and there is no evidence of the involvement of specific lymphocytes [27, 28]. Dental implants are predominantly made of pure titanium (grade 4), but grade 5 titanium alloys and other metal alloys are used in abutments, superstructures and in some implant brands. In the current scientific literature, oral symptoms of titanium intolerance/allergy are imprecisely assigned. Several oral [29–33], but also systemic reactions have been described [30]. In studies on orthopedic hip replacements from alloys, an increased prevalence of metal sensitivity was found in people with metal devices rather than in people without any metal devices (*p* = 0.005) [17, 18]. Patients with a failed implant requiring revision surgery should be considered at risk for hyper-sensitivity to an implant component [19]. There are mainly the following diagnostic tests available: the ECT, MELISA or LTT. The ECT is used as an in-vivo standard test to determine contact sensitization for metals. Immunologically, the mucosa and epidermis differ [34–38] and comparative provocation tests show that necessary allergen concentrations must be 5–12 times higher to trigger a mucosal reaction [39]. The ECT essentially reveals previous epidermal contact sensitization and titanium dioxide 10% was often used as a test allergen [40]. Most patients already have had direct dermal contact to titanium dioxide, as it is contained in multiple products of daily use [41]. The reported test results regarding titanium itself are very heterogeneous with respect to sensitivity and specificity [30, 42–45]. With a few exceptions, allergic contact dermatitis or stomatitis is a type IV allergy according to the classification of Coombs and Gell [46]. Metal ions bind to the body's proteins and function as haptens. In this cell-mediated immune response, antigen-specific CD4 + Th1 lymphocytes and analogous CD8 + Tc1 lymphocytes react with specific antigens. In the oral mucosa, the onset of inflammatory reactions is seen within 24–72 h and the sensitization is referred to as late-type reaction [47]. Due to the salivary “rinse-off” effect and a smaller number of antigen presenting Langerhans cells oral sensitizations are less frequent compared to dermal ones [48].

This review aims to validate the sensitivity and specificity of available diagnostic tests for titanium hypersensitivity and provides recommendation for cases with clinically suspected intolerance reactions to dental titanium implants. Therefore, the following PICO question was formulated: “In Patients with titanium dental implants (P) does epicutaneous testing (ECT) (I), compared to lymphocyte transformation test (LTT) or Memory Lymphocyte Immunostimulation Assay (MELISA) (C) detect hypersensitivity reactions (O)?”.

## Materials and methods

### Protocol development and eligibility criteria

A detailed protocol was developed according to the Preferred Reporting Items for Systematic Review and Meta-Analyses (PRISMA) statement [49]. The following focused question in the Patient, Intervention, Comparison and Outcome (PICO) format was evaluated [50]: “In Patients with titanium dental implants (P) does epicutaneous testing (ECT) (I), compared to lymphocyte transformation test (LTT) or Memory Lymphocyte Immunostimulation Assay (MELISA) (C) detect hypersensitivity reactions (O)?”.

### Inclusion criteria

For the present review, it was decided to include randomized controlled clinical trials, prospective clinical trials as well as retrospective studies presenting diagnostic tests regarding titanium hypersensitivity. The following detailed inclusion criteria were defined:

- Inclusion of greater than ten subjects
- Published in English or German
- Prospective studies: randomized controlled, non-randomized controlled, cohort studies
- Retrospective studies: controlled, case–control, “single cohort”

Studies that did not meet all the above-mentioned inclusion criteria were excluded.

### Exclusion criteria

Literature regarding hypersensitivity reactions in orthopedic implants, hypersensitivity reactions regarding implants not related to dental or maxillofacial surgery, animal studies and in vitro studies were excluded.

### Search strategy

For the comprehensive search strategy, three electronic databases were screened for suitable publications. These sources included the National Library of Medicine, Washington, D. C. (MEDLINE PubMed), the Cochrane Library, and the Scientific Electronic Library Online (SciELO). All three databases were screened for suitable studies until October 5th, 2021. The literature research was completed using the following MeSH Terms (medical subject heading): “titanium” [MeSH] OR “titanium implants” [MeSH] AND “hypersensitivity” [MeSH] OR “allergy” [MeSH], OR “titanium” [MeSH] OR “titanium implants” [MeSH] OR “titanium salts” [MeSH], “hypersensitivity” [MeSH] OR “allergy” [MeSH] OR “titanium intolerance” AND “ECT” [MeSH] OR “Patch Test” [MeSH] OR “LTT” [MeSH] OR “MELISA” [MeSH]. A specific literature research was added to the above-mentioned literature research regarding genetic inflammatory predisposition using “genetic inflammatory predisposition” [MeSH] OR “pro-inflammatory cytokines” AND “implant failure” [MeSH].

### Quality and risk of bias assessment of selected studies

A quality assessment of all selected full-text articles was performed. Randomized, controlled trials were evaluated with the Cochrane Risk of Bias Tool I. Cohort studies were assessed according to the New Castle–Ottawa Scale (NOS) [51, 52] and case series according to Moga et al. 2012 [53].

## Results

### Study selection

**Fig. 2:**
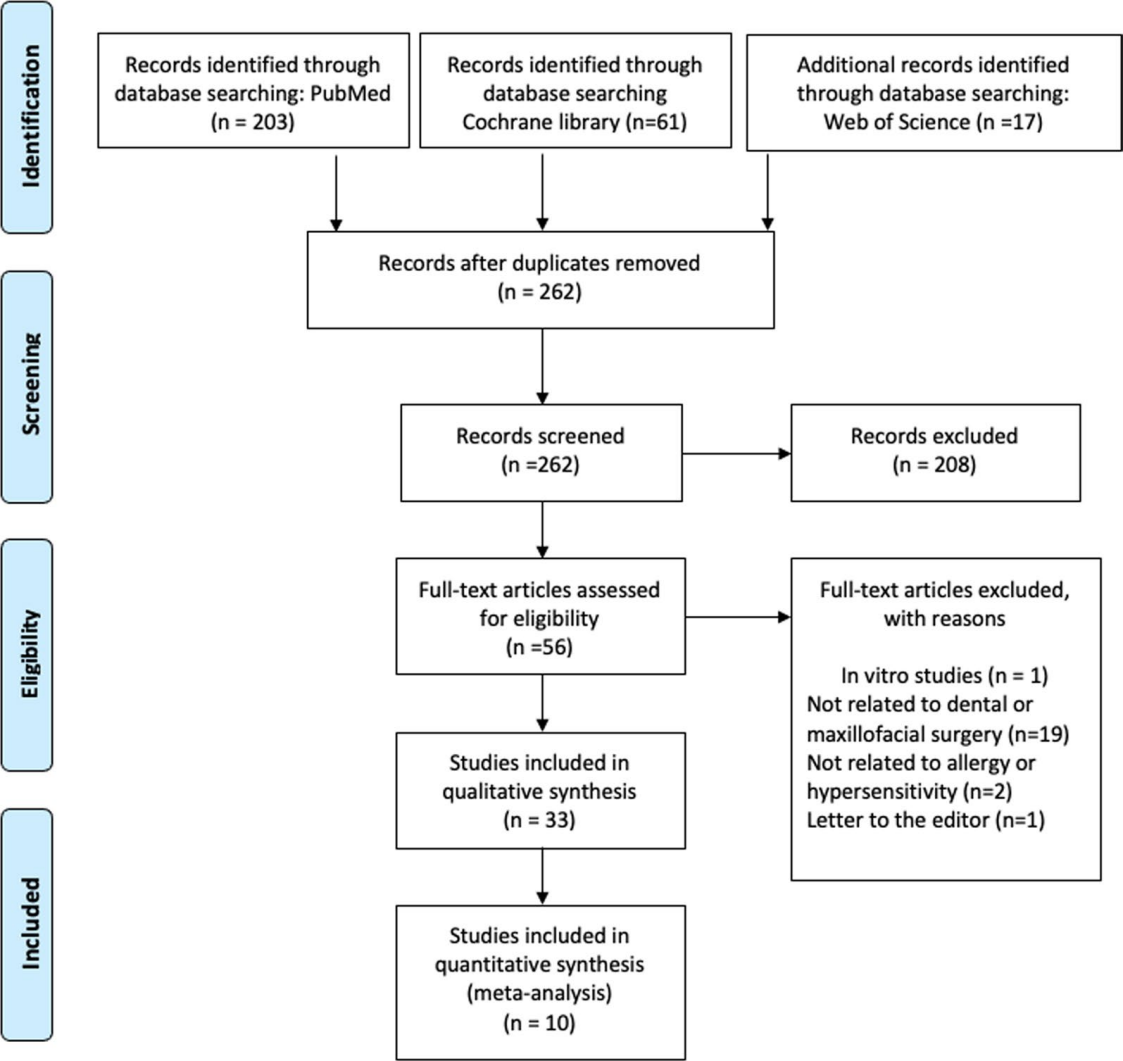
PRISMA flow diagram

The PRISMA flow diagram illustrates the flow of information through the different phases of the systematic review (Fig. 2). It depicts the number of records identified, included, and excluded and the reasons for exclusions. In a first search, there was obvious evidence that no prospective randomized studies could be found on the defined PICO question. Therefore, the possibly best available external evidence was described. The authors are aware that the risk of bias is higher compared with other reviews including only randomized studies. Therefore, drawing definitive conclusions from this data is not recommended.

### Evaluation of study quality and risk of bias

10 studies were included in the quantitative synthesis: 2 clinical studies, 7 cohort studies and 1 case series. (A) The potential for bias (internal validity) for these studies was overall rated as high.

### ECT as diagnostic test for titanium hypersensitivity

In the literature, various titanium salts were used as testing agents. Table 1 summarizes ECT results regarding titanium salts. Whether the titanium salts used in previous tests and studies are present in dental implants has not yet been demonstrated. De Graaf et al. used different titanium salts for epicutaneous testing in a retrospective cohort study. Patients with suspected titanium allergy were tested (*n* = 248; 8.9% positive reaction in ECT), patients with metal allergy to metals other than titanium (*n* = 163, 1.2% positive in ECT), and a control group (*n* = 47, 4.3% tested positive in ECT). Subjects most frequently reacted positively to Ti (IV) oxalate hydrate (7.9%; 216 subjects tested), Ti lactate (4.4%; 45 subjects tested), Ti (IV) isopropoxide (2.9%, 272 subjects tested), Ti citrate (2.2%; 45 subjects tested). Titanium dioxide elicited the fewest positive reactions (0.9%; 329 subjects tested) [43]. In a study by Holgers et al., a total of 18 patients with titanium implants in the head and neck region were included, 9 patients had a clinical history of titanium allergy and 9 patients served as controls without any clinical titanium intolerance [42]. None of the 18 patients reacted positively to ECT performed with a titanium peroxy-gel in different concentrations containing up to 45 mM free titanium [54]. Contamination with *Staphylococcus aureus* was found in 6 of 9 implants of the patients with intolerance reactions, whereas only 2 of 9 implants were contaminated in the control group. In addition, the majority of patients in the test group had a history of psoriasis and seborrheic eczema, but none in the control group. Sicilia et al. asked a patient collective of 1500 patients regarding anamnestic symptoms of titanium intolerance (allergic symptoms after previous implant insertion, unexplained implant loss, soft tissue hyperplasia, previously known metal allergies or high exposure to titanium wear. Thirty-five patients were classified as positive for titanium intolerance. Nine of these 35 patients showed titanium intolerance in ECT (25.7%), and a control group consisting of 35 patients without symptoms showed a prevalence of 0% in ECT. The test substances used were 0.1% and 5% titanium oxide in petrolatum and 0.1% and 5% metallic titanium in an aqueous solution [45]. Hosoki et al. tested 270 patients with intolerance to dental metals, 16 of these patients had dental implants, 68.8% of them showed sensitization reactions to different metals, but only 4 patients showed positive reactions to titanium. The test was performed with 10% titanium dioxide [55]. In a study by Sun et al., 207 patients showed a prevalence of 5.31% with intolerance reactions in cranioplasties using titanium plates. Allergy to more than 3 different metals was associated with a higher risk of osteosynthesis failure. In this study, titanium allergy was not identified [56].

**Table 1:** List of included studies related to titanium allergy tests

Author	Year	Study type	Allergen	Number of patients	Number of patients with positive patch test reaction	Positive patch test reaction [%]	Negative patch test reaction [%]
De Graaf et al.	2018	Retrospective chart review	Titanium	458	26	5.7	94.3
			Ti dioxide	329	3	0.9	99.1
			Ti(IV) isopropoxide	272	8	2.9	97.1
			Ti(IV) oxalate	216	17	7.9	92.1
			Ti lactate	45	2	4.4	95.6
			Ti citrate	45	1	2.2	97.8
Holgers et al.	1992	Clinical trial	Titanium	18	0	0	100
Sicilia et al.	2008	Clinical cohort study	Titanium	35	9	25.7	74.3
Hosoki et al.	2018	Cohort study	Ti dioxide	270	4	1.5	98.5
Sun et al.	2018	Prospective cohort study	Ti chlorid	207	0	0	100
Müller et al.		Cohort study	Ti(IV) oxid	56	0	0	100

### MELISA/LTT as a diagnostic test for titanium hypersensitivity

Müller et al. identified 56 patients with dental implants, dentures or orthodontic brackets, who had a history of intolerance to titanium. In ECT, none of 54 patients tested showed a positive reaction (test substance titanium IV oxide 0.1%), in MELISA, 21 (37.5%) of 56 patients showed a positive reaction to titanium, 16 (28.6%) showed a questionable reaction to titanium, and 19 (33.9%) showed a negative reaction to titanium [57].

Jacobi-Gresser et al. performed a case–control study in 109 patients, *n* = 41 patients with implant loss and *n* = 68 patients with healthy titanium implants served as controls. None of the patients with implant failure showed enhanced T-lymphocyte proliferation to titanium, vanadium or aluminum compared to healthy controls. Although, 6 of 41 patients and 5 of 68 controls were tested positive for nickel sensitization.

## Discussion

**Fig. 3:**
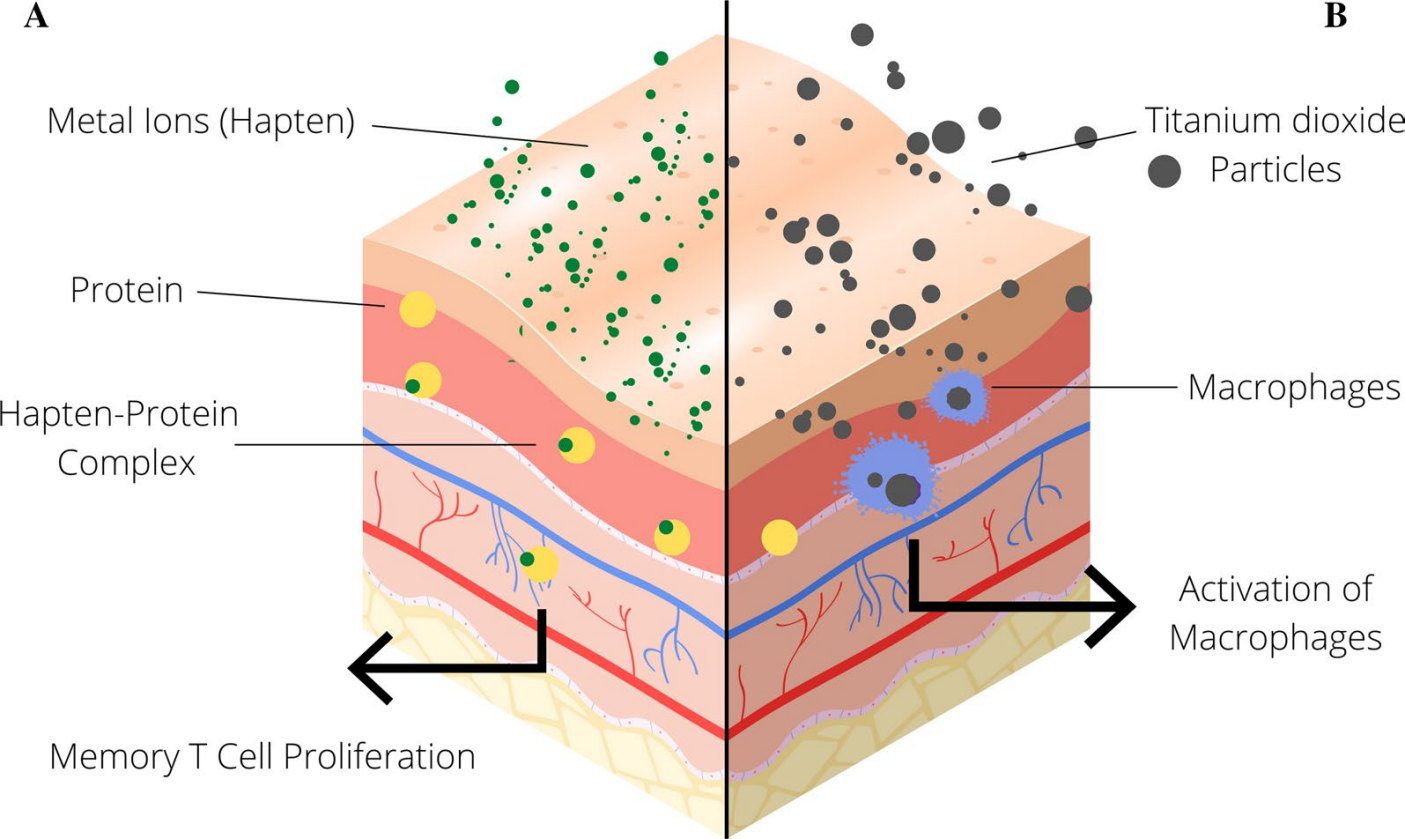
Immunological mechanism of contact hypersensitivity (Coombs and Gell Type IV) as detected by LTT or ECT (**A**). Oxides, such as titanium dioxides, do not react with proteins and, therefore, do not act as haptens. Titan particles stimulate tissue macrophages (**B**)

In the literature, there is the special aspect that ECT is regularly used to test for titanium hypersensitivity [42, 43, 45, 55, 57]. However, ECT with respect to pure titanium, pathophysiologically is not appropriate, because the underlying pathomechanism for a type IV allergy does not exist in titanium, since TiO_2_ cannot act as hapten (Fig. 3). In the case of suspected allergic contact dermatitis of the oral mucosa to other dental prosthetic materials found in implant alloys or in superstructures, such as aluminum, palladium, vanadium, nickel or niobium, the ECT may be effective. In addition, in each individual case the difficult question remains to be answered whether the proven sensitization is pathogenetically causing the intolerance reaction against the endosseous implant. Therefore, the significance of test results for clinical practice must be critically evaluated. Since titanium is highly reactive with oxygen, a passivation layer is built within milliseconds and titanium dioxide is formed on the implant surface. In epicutaneous testing for titanium dioxide, the exposure of the population to the ubiquitous occurrence of TiO_2_ in cosmetics has to be kept in mind and it must be noted that TiO_2_ is poorly soluble and the penetration capacity through the intact cutis is, therefore, suboptimal [45, 57].

The review of the available literature regarding MELISA or LTT in patients with suspected titanium hypersensitivity showed inconsistent results in terms of reliability and validity. The interaction of T-lymphocytes with metal ions determines the basis of the MELISA/LTT assay. In vitro T-cell proliferation assay (LTT) was improved to elicit antigen specific stimulation and decrease bystander proliferation as recognized in MELISA assays especially in titanium testing [58]. Both tests detect T-lymphocytes that had contact with a sensitizing allergen in vitro. Since TiO_2_ does not act as hapten, the significance of test results must be critically evaluated.

A clusterization phenomenon has been observed, since more than half of the overall implant failures occurred in only one-third of the patients, indicating an individual susceptibility to implant failure and implying the existence of genetic risk factors [59]. Cytokines are known to play a key role in modulation of immune reactions. Gene variants of cytokines have been identified to contribute to inflammatory diseases and thus, are known to have an effect on the development of periodontitis and dental implant loss. In accordance with the field of chronic periodontitis, the pro-inflammatory cytokines of the interleukin-1 (IL-1) cluster (IL-1a, IL-1b) are currently the most frequently analyzed factors in peri-implantitis [60]. Gene variants of IL-1a, IL-1b and interleukin 1 receptor antagonist gene (IL-1RN) can result in a higher pro-inflammatory response. A single nucleotide polymorphism (SNP) of pro-inflammatory mediator genes may contribute to their expression levels or amino acid sequence and, consequently, to the host inflammatory response. Stimulation of bone resorption and inhibition of bone formation can be the result of excessive pro-inflammatory activities [61, 62]. Endogenous predispositions may be mediated by host innate immune response to microbial infection and foreign bodies, such as dental implants or cement residues [63]. Oral microbial adhesions at the implant surface and titanium particles from the implant`s oxide layer have the potential to stimulate macrophages to release strong pro-inflammatory cytokines such as (interleukin-1) IL-1 and tumor necrosis factor alpha (TNF-a) [18, 64–66]. Under physiological conditions moderate expression of both cytokines is required for the maintenance of low-grade inflammatory response and osseointegration. However, excessive liberation of these mediators contributes to an increased inflammation, disrupting the physiological balance of bone metabolism, and finally disturbing the osseointegration process.

Functionally relevant polymorphisms in the genes of the cytokines mentioned influence the extent of the release of cytokines. Many studies have been conducted to investigate the association of SNPs with implant failure and related conditions within the last two decades. Some have been found to be associated with peri-implantitis and implant loss and, therefore, have been considered a potential genetic risk factor for implant failure. The genetic variants IL-1A-889C/T and IL-1B-3954 contribute to an increased IL-1 synthesis and consequently to an increased inflammatory capacity [67, 68]. A further increase in inflammatory capacity is found with the polymorphism in TNF-a-308G/A. This genetic variant is associated with an up to sevenfold increase in TNF-a expression [69]. While the named polymorphisms lead to an increased release of pro-inflammatory cytokines, the genetic variant IL-1RN-2018 T/C leads to a decreased release of the anti-inflammatory counterpart. Thus, this polymorphism also has a pro-inflammatory effect. The extent of the initial inflammatory response is primarily determined by the aforementioned cytokines, but also the genetic variants of the cytokines IL-6 and IL-10 can cause a significantly increased tendency to inflammation. The polymorphism -592C/A in the IL-10 gene is associated with reduced IL-10 synthesis and as a result with reduced inhibition of inflammation [70]. Significantly increased bone resorption and tissue destruction have been demonstrated in the presence of reduced IL-10 release in affected patients. Genotypes of pro-inflammatory cytokines and gene variants of anti-inflammatory cytokines can, therefore, reinforce or counterbalance the effect on inflammation. Furthermore, the influence of the IL-1 genotypes on implant failure was examined [64], but no significant association could be found in univariate analyses. However, a variable number of tandem repeat IL-1RN gene polymorphisms is more frequent in patients with peri-implantitis [71].

The close association of the four genetic variants IL-1a, IL-1b, TNF-a and IL-1RN with the risk of peri-implantitis development or implant loss has been demonstrated in a large number of worldwide association studies and was also confirmed in meta-analyses [72, 73]. It was concluded that functional polymorphisms of IL-a < (C-889 T), IL-b (C + 3954 T and C-511 T) and composite genotype of IL-1 are valuable as predictive markers for peri-implant disease.

Furthermore, Jacobi-Gresser et al. observed an association between IL1/TNF-a single nucleotide polymorphisms and implant failure independently from smoking status. Whereas increased risk of implant failure could not be observed in existence of single SNPs of IL1, TNF-a and IL-1RN genes, respectively, the occurrence of implant failure proportionally increased with rising numbers of identified risk polymorphisms in the analyzed genes [74]. A number of retrospective studies demonstrated a correlation between polymorphisms of interleukins IL-1a, IL-1b, IL-1RN, IL-6, IL-10 as well as TNF-a and the incidence of periodontitis and peri-implant disease [75]. The clinical relevance of the aforementioned polymorphisms is also demonstrated by the fact that patients with risk polymorphisms for IL-1 and TNF-a show increased susceptibility to peri-implant bone loss [76], especially in combination with toxic environmental factors, such as smoking [77, 78]. Another promoting influence for chronic inflammatory diseases such as periodontitis and peri-implantitis is associated with increased IL-6 expression [79–81].

The individual risk for titanium implant-associated peri-implantitis is strongly dependent on genetic susceptibility, analogous to periodontitis, and is often additionally triggered by titanium particle load in the peri-implant tissue. As known from other complex inflammatory diseases, the clinical impact is not based on the presence of one single gene variant only, but it is associated with the presence of polymorphisms of multiple functional genes involved in peri-implant disease development. Risk profiling with the evaluation of genetic predisposition to inflammation may be a valuable option for individual risk assessment prior to implant insertion.

## Conclusions

The review of the available literature regarding ECT and MELISA or LTT in patients with suspected titanium hypersensitivity showed inconsistent results in terms of reliability and validity. Due to the extremely high potential for oxidation, titanium oxidizes rapidly and per definition can no longer act as hapten. Therefore, it is not possible to induce a type IV allergy as detected by ECT, LTT or MELISA. In consequence these tests should not be performed in patients with pure titanium implants and suspected titanium intolerance reaction.

**Fig. 4:**
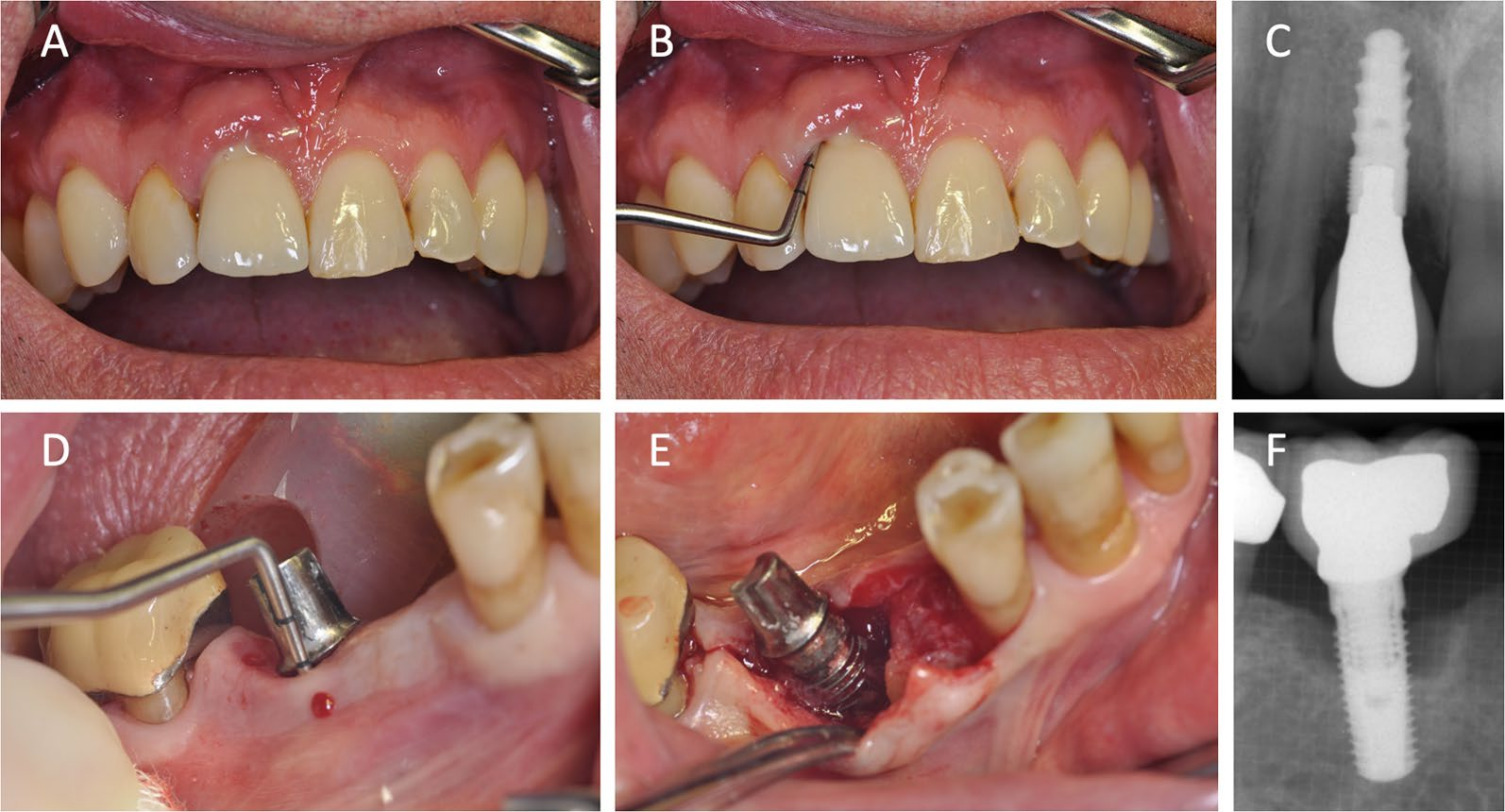
Intraoral aspect of suspected intolerance/hypersensitivity to titanium implant placed in the upper incisor region in a patient without periodontal risk predisposition 2 years after implant insertion (**A** and **B**) and corresponding intraoral radiograph (**C**) and in the lower first molar region in a patient with genetic pro-inflammatory predisposition 7 years after implant surgery (**D** and **E**) and corresponding intraoral radiograph (**F**)

New diagnostic methods and research about the complex phenomenon on hypersensitivity reaction regarding titanium are needed. It should be kept in mind, that titanium alloys contain aluminium and vanadium and may be contaminated with nickel.Furthermore, superstructures may contain a variety of metals beyond titanium, which may act as haptens. Genetic predispositions for hyper-inflammatory reactions may be independent individual risk factors. Immunological mechanisms of the breakdown of the foreign body equilibrium are not yet fully elucidated and clinical presentation of titanium particle induced hypersensitivity compared to biofilm associated inflammatory peri-implantitis may be hard to distinguish (Fig. 4). Due to the bacterial invasion, immunological pathways may even overlap in both, peri-implantitis and hypersensitivity reactions [18]. Thus, clinical diagnostic of a titanium hypersensitivity is complex. Therefore, the authors recommend peri-implant therapy and removing of any potentially allergy-related suprastructures in the first line, since they may contain grade 5 titanium alloys and other metal alloys before considering removal of the endosseous dental implant due to suspected hypersensitivity reactions. More scientific research is needed to explain the pathophysiological phenomenon of titanium hyper-sensitivity and to develop valid and reliable assays. As far as we understand by now, titanium sensitivity/titanium intolerance is a hyper-reactivity of macrophages (monocyte cell line) to titanium nano-and microparticles. There are functional immune tests introduced to the market, which need to be verified by further clinical trials.

## Data Availability

Not applicable.
